# FTO interacts with FOXO3a to enhance its transcriptional activity and inhibits aggression in gliomas

**DOI:** 10.1038/s41392-020-00234-3

**Published:** 2020-07-28

**Authors:** Bangbao Tao, Xuehua Huang, Juanhong Shi, Jun Liu, Shu Li, Chunyan Xu, Jun Zhong, Liang Wan, Baohui Feng, Bin Li

**Affiliations:** 1 Department of Neurosurgery, Xinhua Hospital, School of Medicine, Shanghai Jiaotong University, 1665 Kongjiang Road, 200092 Shanghai, China; 2 Department of Pain, Xinhua Hospital, School of Medicine, Shanghai Jiaotong University, 1665 Kongjiang Road, 200092 Shanghai, China; 3 Department of Pathology, Tongji Hospital, School of Medicine, School of Medicine, Shanghai Tongji University, 389 Xincun Road, 200062 Shanghai, China; 4 Department of Neurosurgery, Second Affiliated Hospital of Wannan Medical College, 10 Kangfu Road, 241001 Wuhu, Anhui China; 5 Department of Pathophysiology, Wannan Medical College, 22 Wenchang Road, 241002 Wuhu, Anhui China

**Keywords:** CNS cancer, CNS cancer

**Dear Editor**,

Gliomas represent the most common primary brain tumors in adults and few targeted therapies are available. Thus, it’s imperative to explore novel target genes in gliomas. Fat mass and obesity-associated (FTO) gene encodes an alpha-ketoglutarate-dependent dioxygenase, which demethylates N6-methyladenosine on single-strand RNA.^1^ FTO is highly expressed in the brain but its potential contribution to gliomas is unknown. FOXO3a belongs to the forkhead family of transcription factors and it mediates important cellular processes like apoptosis and proliferation by regulating its target gene expression.^2^ Interestingly, genetic variants of FOXO3^3^ and FTO^4^ are associated with longevity in human, suggesting that FTO and FOXO3a might share convergent effects in biological processes. Thus, we investigated the involvement of potential FTO–FOXO3a interaction in gliomas.

First, we measured FTO protein level in clinical samples of gliomas (*n* = 50) and normal brain tissues (*n* = 7) by western blot and immunochemistry. Western blot (Fig. 1a) showed that FTO protein levels were downregulated in gliomas compared to normal tissues. This was further confirmed by immunochemistry in sections from the same samples. The results showed that FTO staining produced strong nuclear and moderate cytoplasmic signals in neurons and glial cells in normal brain tissue, but much weaker signal in glioma tissues (Fig. 1b). Quantification of FTO staining by Quickscore showed that both the positive rate and Quickscore were reduced in gliomas compared to normal tissues (Supplementary Fig. S1a). In addition, FTO was further reduced in high-grade gliomas compared to low-grade gliomas. FTO mRNA level was also measured by qPCR and it showed a downregulation of FTO mRNA level in gliomas compared to normal tissues (Supplementary Fig. S1b). This was cross-validated by expression data from TCGA GBM (total *n* = 454, Fig. S1c) and REMBRANDT glioma (total *n* = 524, Supplementary Fig. S1d) datasets. Kaplan–Meier survival analysis showed patients with low FTO expression had significantly shorter survival time compared to those with high FTO expression in REMBRANDT gliomas (HR = 0.71, *P* = 0.0047, Supplementary Fig. S1e) and TCGA GBM (HR = 0.82, *P* = 0.045, Supplementary Fig. S1f) datasets. FOXO3a staining in the sections of gliomas showed that the nearly all FOXO3a signals were accumulated in the nucleus in low-grade gliomas while high-grade gliomas had less nuclear localization of FOXO3a (Fig. 1c). It suggests that FTO reduction is associated with less nuclear localization of FOXO3a in gliomas.

**Fig. 1:**
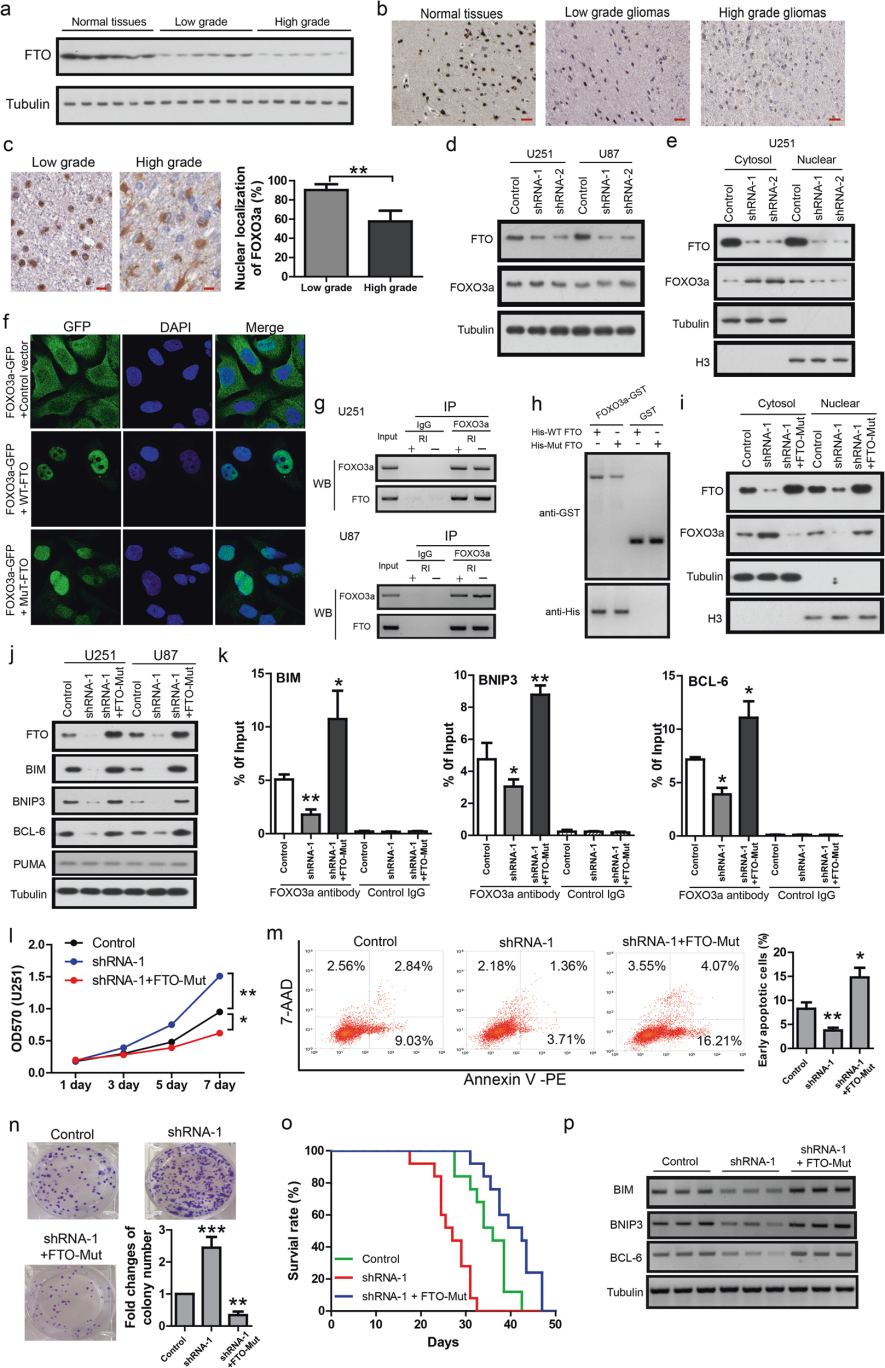
**a** Representative western blot showing FTO protein levels in normal brain tissues (*n* = 7), low-grade gliomas (*n* = 20) and high-grade gliomas (*n* = 30). **b** Representative images of FTO staining in normal tissues, low-grade and high-grade gliomas. Scale bar = 60 µm. **c** Representative images of FOXO3a staining in low-grade and high-grade gliomas. Scale bar = 25 µm. **d** Representative western blots showing the protein levels of FTO and FOXO3a in the presence of FTO knock-down in U251 and U87 cell lines. **e** Representative western blots showing the subcellular distribution of FTO and FOXO3a in the presence of FTO knock-down in U251 cell. **f** Immunofluorescence of U251 cells transfected with FOXO3a-GFP together with control, WT-FTO or Mut-FTO. **g** Representative western blots showing the precipitated protein levels of endogenous FOXO3a and FTO in U251 and U87 cell lines in the presence or absence of RNase inhibitor (RI). **h** GST pull-down of WT or Mut FTO protein by FOXO3a–GST protein. **i** Representative western blots showing the subcellular distribution of FTO and FOXO3a in the presence of control, FTO shRNA-1 or FTO shRNA-1 plus FTO-Mut over-expression in U251 cell. **j** Representative western blots showing the protein levels of FTO, BIM, BNIP3, BCL-6 and PUMA in U251 and U87 infected with control, FTO shRNA-1 and FTO shRNA-1 plus FTO-Mut. **k** ChIP-qPCR results showing FOXO3a binding on the promoters of BIM, BNIP3, and BCL-6, respectively. **l** MTT assay showing the growth curves of U251 infected with control, FTO shRNA-1 and FTO shRNA-1 plus FTO-Mut over-expression. **m** Representative results and quantification of Annexin V-PE apoptosis assay in U251 infected with control, FTO shRNA-1 and FTO shRNA-1 plus FTO-Mut over-expression. **n** Representative images and quantification of colony formation of U251 infected with control, FTO shRNA-1 and FTO shRNA-1 plus FTO-Mut over-expression. **o** Kaplan–Meier survival curves of nude mice bearing intracranial gliomas infected with control, FTO shRNA-1 or FTO shRNA-1 + FTO-Mut. **p** Western blot results showing the protein levels of BIM, BNIP3 and BCL-6 in intracranial glioma tissues infected with control, FTO shRNA-1 or FTO shRNA-1 + FTO-Mut. For all, data were presented as mean ± SD. *P < 0.05; **P < 0.01; ***P < 0.001

To investigate the potential effects of FTO on FOXO3a, FTO knock-down was achieved with two independent lenti-viral shRNAs in two glioma cell lines (U251 and U87) and it had no effect on FOXO3a protein level (Fig. 1d). Similarly, lenti-viral over-expression of wild-type FTO (FTO-WT) or its mutant form lacking enzyme activity (FTO-Mut) (Supplementary Fig. S2a) had no effect on FOXO3a protein level (Supplementary Fig. S2b). As FOXO3 shuttles between nucleus and cytoplasm, we measured the potential effect of FTO on the subcellular distribution of FOXO3a. It shows that FTO knock-down reduced nuclear accumulation of FOXO3a in U251 cell (Fig. 1e) and U87 cell (Supplementary Fig. S2c). FTO-WT or FTO-Mut over-expression increased nuclear accumulation of FOXO3a (Supplementary Fig. S2d). We also measured the trafficking of FOXO3a in U251 cell using a GFP tagged FOXO3a construct (Fig. 1f). In FOXO3a–GFP transfected cells, FOXO3a–GFP was mainly localized in the cytosol and co-transfection of FTO–WT or FTO-Mut resulted in dramatic nuclear translocation of FOXO3a–GFP. These results suggest that FTO could enhance the nuclear translocation of FOXO3a independent of FTO enzyme activity in glioma cells. As FTO shuttles between the nucleus and cytoplasm and previous study shows that FTO directly interacts with another transcriptional factor-CaMKII,^5^ we hypothesized that FTO may interact with FOXO3a to facilitate its nuclear translocation. Co-immunoprecipitation was performed in the presence or absence of RNase inhibitor to detect FTO–FOXO3a interaction in U251 and U87 cells. The results show that FTO was co-precipitated with FOXO3a in both cell lines and treatment of RNase inhibitor had no effect on FTO–FOXO3a interaction. It suggests that FTO may have non-overlapping domains for FOXO3a interaction and RNA binding, respectively (Fig. 1g). To investigate whether FTO–FOXO3a interaction is dependent on its enzyme activity, we over-expressed HA-tagged FOXO3a together with Myc-tagged FTO-WT or FTO-Mut in HEK293 cell. Reciprocal Co-immunoprecipitation shows that FOXO3a-HA was co-precipitated with FTO-WT-myc or FTO-Mut-myc, and both FTO-WT-myc and FTO-Mut-myc were co-precipitated with FOXO3a-HA (Supplementary Fig. S3a). To confirm the direct interaction between FTO and FOXO3a, we purified recombinant WT or Mut FTO proteins with His tag and their purity was confirmed by silver staining (Supplementary Fig. S3b). GST pull-down was performed with recombinant FOXO3a-GST and His tagged FTO-WT or FTO-Mut. The results showed that FOXO3a–GST could pull-down FTO-WT and FTO-Mut (Fig. 1h). Thus, both FTO-WT and FTO-Mut can directly interact with FOXO3a. In addition, FTO-Mut reversed the cytoplasmic accumulation of FOXO3a induced by FTO knock-down in U251 (Fig. 1i) and U87 (Supplementary Fig. S3c) cells. Taken together, these results support that FTO interacts with FOXO3a to facilitate its nuclear translocation.

Next, we measured the potential effects of FTO on several well-established target genes of FOXO3a, including BIM, BNIP3, BCL-6, and PUMA. Western blot (Fig. 1j) and qPCR (Supplementary Fig. S4a) showed that FTO knock-down reduced the expression of BIM, BNIP3, and BCL-6 and this was fully reversed by FTO-Mut over-expression. Consistently, both FTO-WT and FTO-Mut increased the expression of BIM, BNIP3 and BCL-6 (Supplementary Fig. S4b). To confirm that FTO induced the above gene expression changes through FOXO3a, ChIP-qPCR was performed in U251 cell. The results showed that FTO knock-down resulted in less FOXO3a binding on the promoters of BIM, BNIP3, and BCL-6 while FTO-Mut fully rescued these effects (Fig. 1k). As FTO might modulate FOXO3a target genes to regulate tumor behaviors, we investigated the potential effects of FTO in in vitro and in vivo assays. MTT assay showed that FTO knock-down promoted cell proliferation and FTO-Mut reversed this effect in U251 (Fig. 1l) and U87 (Supplementary Fig. S5a). Annexin V-PE apoptosis assay showed that FTO knock-down inhibited apoptosis and FTO-Mut reversed this effect in U251 (Fig. 1m) and U87 (Supplementary Fig. S5b). In colony formation assay, FTO knock-down increased colony numbers and FTO-Mut reversed this effect in U251 (Fig. 1n) and U87 (Supplementary Fig. S5c). In intracranial glioma model of nude mice, mice bearing FTO shRNA-1-infected U251 cells (*n* = 13) had shorter survival time compared to mice bearing Control-infected U251 cells (*n* = 13) (*P* < 0.01). Mice bearing FTO shRNA-1 plus FTO-Mut infected U251 cells (*n* = 13) had longer survival time compared to mice bearing Control-infected U87 cells (*P* < 0.05) or mice bearing FTO shRNA-1-infected U251 cells (*P* < 0.001) (Fig. 1o). It suggests that FTO inhibits in vivo aggression of gliomas. The expression of BIM, BNIP3 and BCL-6 in the intracranial gliomas from the above three groups was measured by qPCR (Supplementary Fig. S6a) and western blot (Fig. 1p). The results showed that the expression of BIM, BNIP3, and BCL-6 was reduced in gliomas from FTO shRNA-1 group compared to Control group while FTO-Mut fully rescued this effect. It suggests that FTO modulated FOXO3a target gene expression in in vivo condition. This was further cross-validated by expression data from TCGA GBM and TCGB LGG datasets which showing positive correlations of FTO with BIM, BNIP3 or BCL-6, respectively (Supplementary Fig. S6b).

In summary, we find that FTO plays a tumor-suppressive role in gliomas by interacting with FOXO3a to enhance its nuclear translocation and target gene expression (Supplementary Fig. S6c). As FTO downregulation is associated with poor prognosis and promotes aggression, restoring the endogenous expression of FTO by methods such as CRISPR-based activation may have therapeutic potential for gliomas.

## Supplementary information

Supplementary information

## Data Availability

All data relevant to this work are included in this paper and its supplementary information.
