# Supplementary material for: FTO interacts with FOXO3a to enhance its transcriptional activity and inhibits aggression in gliomas
Source: Signal Transduct Target Ther. 2020 Jul 28;5:130. doi: 10.1038/s41392-020-00234-3 (PMC7387469; doi:10.1038/s41392-020-00234-3)
Supplement: Supplementary file 1 — Supplementary information [file 41392_2020_234_MOESM1_ESM.docx]

Supplementary Materials for

FTO interacts with FOXO3a to enhance its transcriptional activity and inhibits aggression in gliomas

Bangbao Tao, Xuehua Huang, Juanhong Shi, Jun Liu, Shu Li, Chunyan Xu, Jun Zhong, Liang Wan, Baohui Feng, Bin Li

Correspondence to: Bangbao Tao (taobangbao@xinhuamed.com.cn), Liang Wan (wanliang@xinhuamed.com.cn), Baohui Feng (fengbaohui@xinhuamed.com.cn), Bin Li (Libin@xinhuamed.com.cn)

**This PDF file includes:**

Materials and Methods

Figures. S1 to S6

**Materials and Methods**

1. Clinical samples

The study was approved by the Review Boards of Xinhua Hospital (Shanghai, China) and written informed consent was obtained from each patient. A total of 50 cases of primary glioma tissues were collected in Xinhua Hospital and all cases were confirmed by pathological diagnosis. The Grading of gliomas was performed according to the 2007 WHO Classification criteria. A total of 7 normal brain tissue samples were from non-glioma patients undergoing brain surgery. In this study, all cases of gliomas were classified as low grade (WHO I and II, n=20) or high grade (WHO III and IV, n=30) for statistical analysis.

2. Cell cultures

Glioma cell lines U87 and U251, and HEK293T from ATCC were maintained in Dulbecco’s Modified Essential Medium (DMEM) with 10% fetal bovine serum (FBS), 100 U/ml penicillin and 100 mg/ml streptomycin. Cells were cultured in a humidified atmosphere with 5 % CO2 at 37℃.

3. Constructs for FTO over-expression/knock-down and FOXO3a-GFP

For FTO over-expression, the coding sequence of human FTO (NM_001080432) with C-terminal Myc tag was cloned into pLenti-P2A-tGFP vector. For the FTO mutant with H231A/D233A mutations which disrupt its enzymatic activity, shRNA resistant point mutations were introduced. For FTO knock-down, two the short hairpin RNAs (shRNAs) targeting different sites of human FTO mRNA sequence (shRNA-1 forward: TGGCAGTGTATCTGAGGAGCTCCATAATTCAAGAGATTATGGAGCTCCTCAGATACACTGCCTTTTTTC; shRNA-1 reverse: TCGAGAAAAAAGGCAGTGTATCTGAGGAGCTCCATAATCTCTTGAATTATGGAGCTCCTCAGATACACTGCCA; shRNA-2 forward: TGTCAGCGGTGGCAGTGTACAGTTATATTC AAGAGATATAACTGTACACTGCCACCGCTGACTTTTTTC; shRNA-2 reverse: TCGAGAAAAAAGTCAGCGGTGGCAGTGTACAGTTATATCTCTTGAATATAACTGTACACTGCCACCGCTGACA) and the nonsense control shRNA were constructed into the pLentiLox3.7 (pLL3.7) lentiviral vector. Cell lines were infected at an MOI of 5. For FOXO3a-GFP translocation experiment, WT and Mut FTO were cloned into pcDNA3.1 vector, respectively. A GFP-tagged FOXO3a construct was from Origene (RG209846). These vectors were transfected using lipofectamine 2000.

4. In vitro assays for tumor cell behaviors

MTT cell proliferation assay, colony formation assay and apoptosis assay were performed as previously described^1^. Each experiment was biologically repeated for three times and the data were presented as fold changes relative to control group.

5. Intracranial glioma model

U251 cells were infected with lenti-virus (Control, FTO shRNA-1, FTO shRNA-1 + FTO-Mut) at MOI of 10. One day after infection, 5 × 10^5^ cells were suspended in 5 μL Leibovitz L-15 medium, injected into the right caudate putamen of athymic nude male mice (6 weeks old, n = 13–15 per group) using a 50-μL syringe and 23-gauge needle. Briefly, a 0.5-mm burr hole was made 2 mm right to the midline and 1 mm anterior to the bregma before the stereotactical injection at a depth of 3 mm. The care of athymic nude mice was in accord with the animal welfare guidelines of Xinhua Hospital, Shanghai Jiaotong University.

6. Quantitative Real-time PCR

Total RNA was extracted from tissues or cell lines using TRIzol reagent and quantitative real time-PCR was performed as previously described^1^. Following qRT-PCR primers were used. FTO: ACTTGGCTCCCTTATCTGACC (forward) and TGTGCAGTGTGAGAAAGGCTT (reverse); BIM: TAAGTTCTGAGTGTGACCGAGA (forward) and GCTCTGTCTGTAGGGAGGTAGG (reverse); BNIP3: CAGGGCTCCTGGGTAGAACT (forward) and CTACTCCGTCCAGACTCATGC (reverse); BCL-6: ACACATCTCGGCTCAATTTGC (forward) and AGTGTCCACAACATGCTCCAT (reverse); PUMA GACCTCAACGCACAGTACGAG (forward) and AGGAGTCCCATGATGAGATTGT (reverse); actin: ACCAACTGGGACGACATGGAGAAA (forward) and TAGCACAGCCTGGATAGCAACGTA (reverse).

7. Western blot

Western blot was performed as previously described^1^. Following antibodies were used: FTO (abcam, ab124892), FOXO3a (abcam, ab53287), BIM (abcam, ab32158), BNIP3 (abcam, ab10433), BCL-6 (ab172610) and PUMA (abcam, ab33906), GST (abcam, ab111947), His (CST, 12698), Myc (CST, 2276) and HA (CST, 3724).

8. Co-immunoprecipitation and Preparation of Cytoplasmic and Nuclear Fractions

Cells were lysed in RIPA lysis buffer with complete protease inhibitor cocktail and SUPERase• In RNase Inhibitor. Cell lysates were centrifuged at 15000g for 20 min at 4°C. The supernatant was incubated indicated antibodies or magnetic beads (Pierce Anti-HA Magnetic Bead or Pierce Anti-myc Magnetic Bead) at 4°C overnight. Then, the protein complexes were collected by incubation with Protein A/G beads or magnetic stand. The collected protein complexes were washed with RIPA buffer for 5 times and eluted by SDS loading buffer. The isolation of cytoplasmic and nuclear fractions was performed using Nuclear/Cytosol Fractionation Kit (Biovision, K266-100) according to the manufacturer’s instruction. The samples were analyzed by western blot.

9. Immunochemistry and Immunofluorescence

Immunochemistry analysis was performed blindly as previously described^1^. Following antibodies were used: FTO antibody (1:100 dilution) and FOXO3a (1:300 dilution). Immunofluorescence in cultured cells was performed as previously described^1^ and GFP fluorescence was directly imaged by Nikon Ti microscope.

10. ChIP-qPCR

Chromatin immunoprecipitation (ChIP)-Qpcr was performed as previously described^1^. Following primers were used to detect promoter regions containing potential FOXO3a binding sites for each target gene. BIM: AGGCTAGGGTACACTTCG (forward) and AGGCTCGGACAGGTAAAG (reverse)^2^; BNIP3: CCTCCTTCTAAATTAAGCTGTTCTT (forward) and GGGAGGCTGTTGTAATTCCTC (reverse)^3^; BCL-6: GGCCTTTAGAATTCCCTCCGGC (forward) and GCTGAAGTGTGTCTCTCCTGCAC (reverse) ^4^.

11. Recombinant FTO protein purification and GST pull-down assay

The coding sequences of wild-type and mutant FTO were cloned into PET-28a(+) vector and the expression of His-tagged FTO fusion protein was induced with 1 mM IPTG for 12h at 37 °C in BL21 Escherichia coli (Novagen). The bacteria were collected by centrifugation and the soluble proteins were extracted by BugBuster Master Mix (Novagen) and purified by Ni-column (QIAGEN) and reconstituted in TBS. The purity of wild-type and mutant FTO fusion proteins was analyzed by Pierce Silver Stain Kit (24612). In GST pull-down assay, the purified WT or Mut FTO recombinant protein (1ug) was incubated with 1ug of FOXO3a-GST fusion protein (abcam, ab114191) or GST protein (abcam, ab70456) at 37 °C for 2 hours. GST Pull-down was performed using Pierce™ GST Protein Interaction Pull-Down Kit (21516) according to the manufacturer’s instruction. The samples were analyzed by western blot.

12. m6A RNA Methylation Quantification

The total level of m6A RNA methylation was measured by EpiQuik m6A RNA Methylation Quantification Kit according to the manufacturer’s instruction.

13. Statistical analysis

Statistical analysis was performed using GraphPad Prism software. All data from cell lines were presented as mean ± SD and statistical analysis was performed by two-tailed Student t test for two groups and one way ANOVA with Newman-Keuls post hoc test for more than two groups. All data from clinical samples were presented as whiskers-box plots and non-parametric Mann–Whitney U-test was used for two groups and Kruskal–Wallis test followed by post hoc Dunn’s multiple comparison test was used for more than two groups. Analysis of expression data and survival data in REMBRANDT and TCGA datasets was performed as previously described^5^. Statistically significant differences were defined as P < 0.05. For all, *P<0.05, **P<0.01, ***P<0.001.

**Reference**

1 Li, S. *et al.* HOXC10 promotes proliferation and invasion and induces immunosuppressive gene expression in glioma. *Febs J* **285**, 2278-2291, doi:10.1111/febs.14476 (2018).

2 Czymai, T. *et al.* FOXO3 Modulates Endothelial Gene Expression and Function by Classical and Alternative Mechanisms. *J Biol Chem* **285**, 10163-10178, doi:10.1074/jbc.M109.056663 (2010).

3 Lin, A. *et al.* The FoxO-BNIP3 axis exerts a unique regulation of mTORC1 and cell survival under energy stress (vol 33, pg 3183, 2014). *Oncogene* **33**, 5310-5310, doi:10.1038/onc.2014.330 (2014).

4 de Mattos, S. F. *et al.* FoxO3a and BCR-ABL regulate cyclin D2 transcription through a STAT5/BCL6-dependent mechanism. *Mol Cell Biol* **24**, 10058-10071, doi:10.1128/Mcb.24.22.10058-10071.2004 (2004).

5 Tao, B. B. *et al.* CA10 and CA11 negatively regulate neuronal activity-dependent growth of gliomas. *Mol Oncol* **13**, 1018-1032, doi:10.1002/1878-0261.12445 (2019).


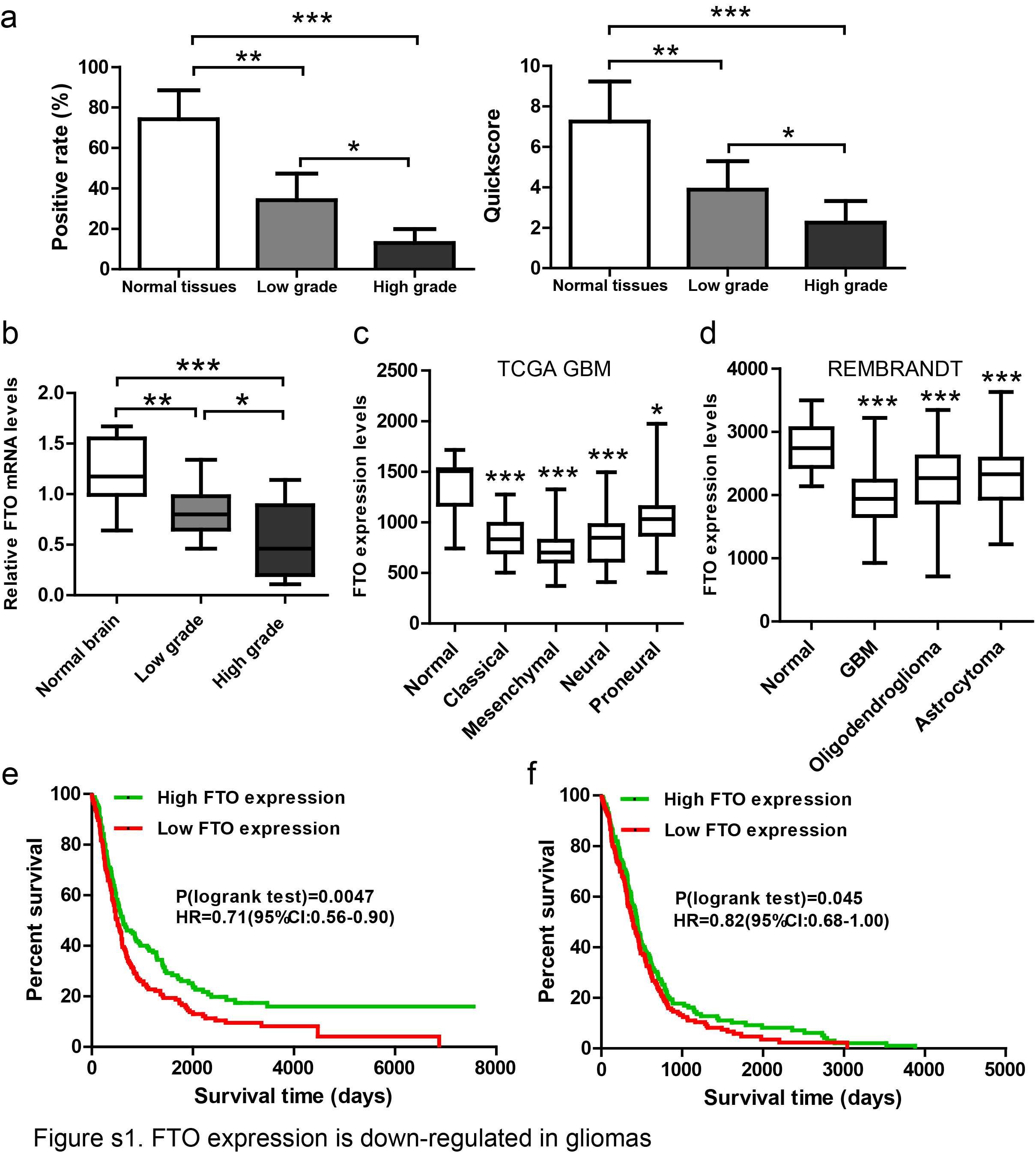


Figure s1. FTO expression is down-regulated in gliomas

(a) Positive rate and Quickscore of FTO staining in normal brain tissues (n=7), low-grade gliomas (n=20) and high-grade gliomas (n=30). (b) qPCR results showing FTO mRNA levels in the same samples. Data were presented as whiskers-box plots. (c) FTO expression data in TCGA GBM dataset (total n=454). (d) FTO expression data in REMBRANDT glioma dataset (total n=524). Kaplan-Meier survival curves of patients classified by FTO expression in REMBRANDT gliomas dataset (HR=0.71, P=0.0047) (e) and TCGA GBM dataset (HR=0.82, P=0.045) (f). For all, *P<0.05; **P<0.01; ***P<0.001.


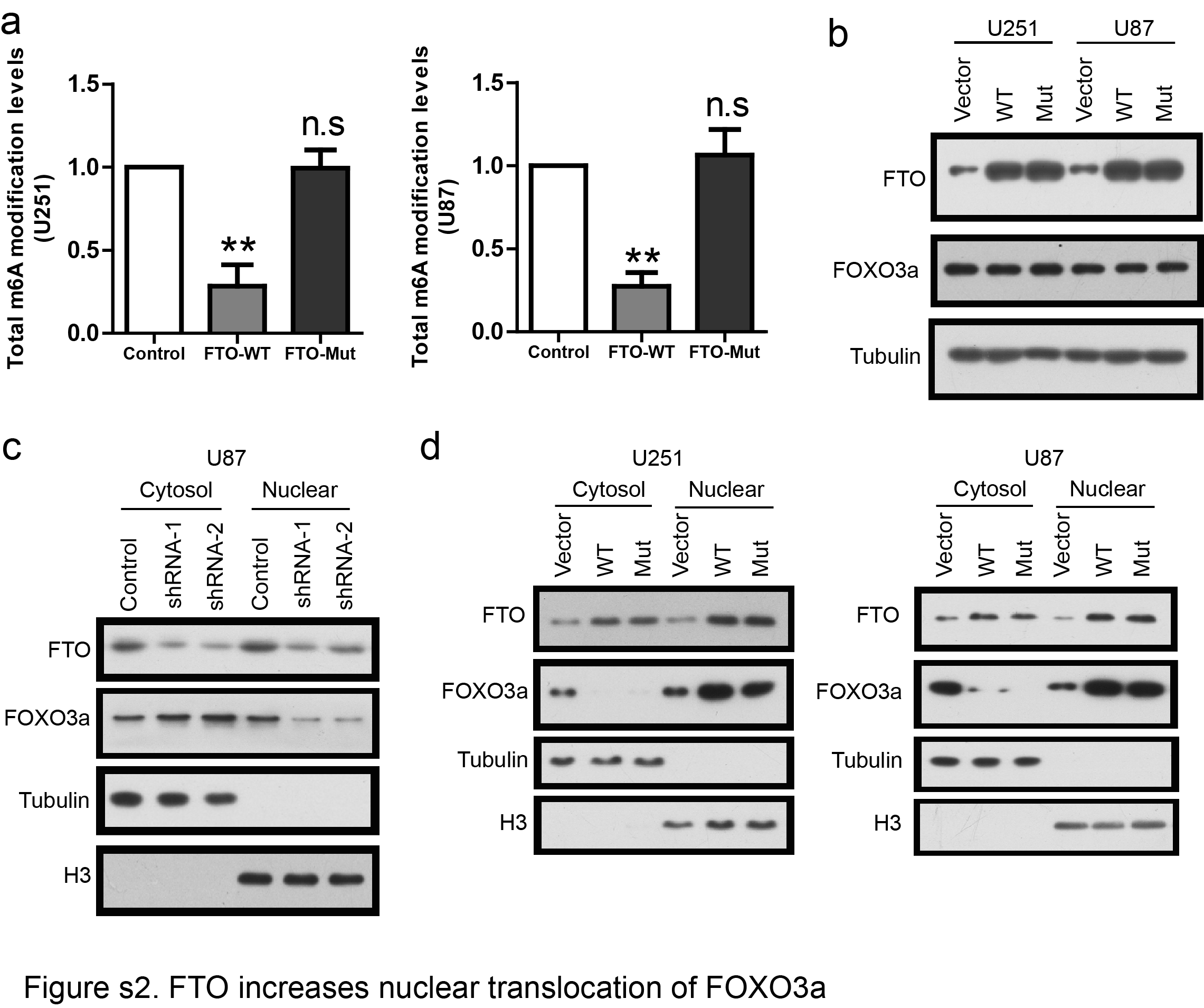


Figure s2. FTO increases nuclear translocation of FOXO3a

(a) Quantification of m6A modification levels in U251 and U87 cell lines infected with indicated lentivirus. (b) Representative western blots showing the protein levels of FTO and FOXO3a in the presence of FTO-WT or FTO-Mut over-expression in U251 and U87 cell lines. (c) Representative western blots showing the subcellular distribution of FTO and FOXO3a in the presence of FTO knock-down in U87 cell line. (d) Representative western blots showing the subcellular distribution of FTO and FOXO3a in the presence of FTO-WT or FTO-Mut over-expression in U251 and U87 cell lines.


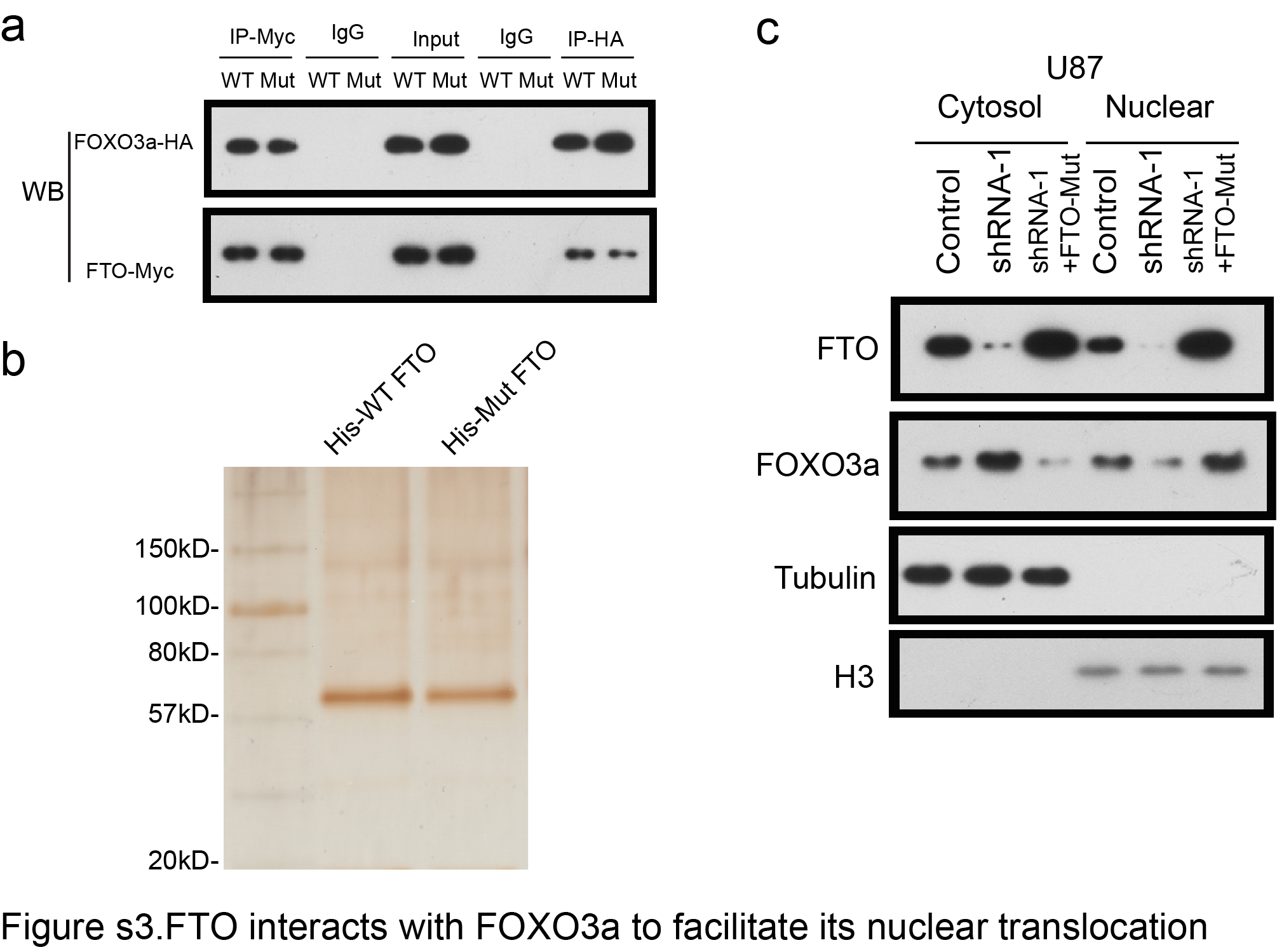


Figure s3. FTO interacts with FOXO3a to facilitate its nuclear translocation

(a) Representative western blots showing reciprocal Co-immunoprecipitation of over-expressed HA-tagged FOXO3 and Myc-tagged FTO-WT or FTO-Mut in HEK293 cell. (b) Silver staining of purified His-WT FTO and His-Mut FTO proteins. (c) Representative western blots showing the subcellular distribution of FTO and FOXO3a in the presence of control, FTO shRNA-1 or FTO shRNA-1 plus FTO-Mut over-expression in U87 cell line.


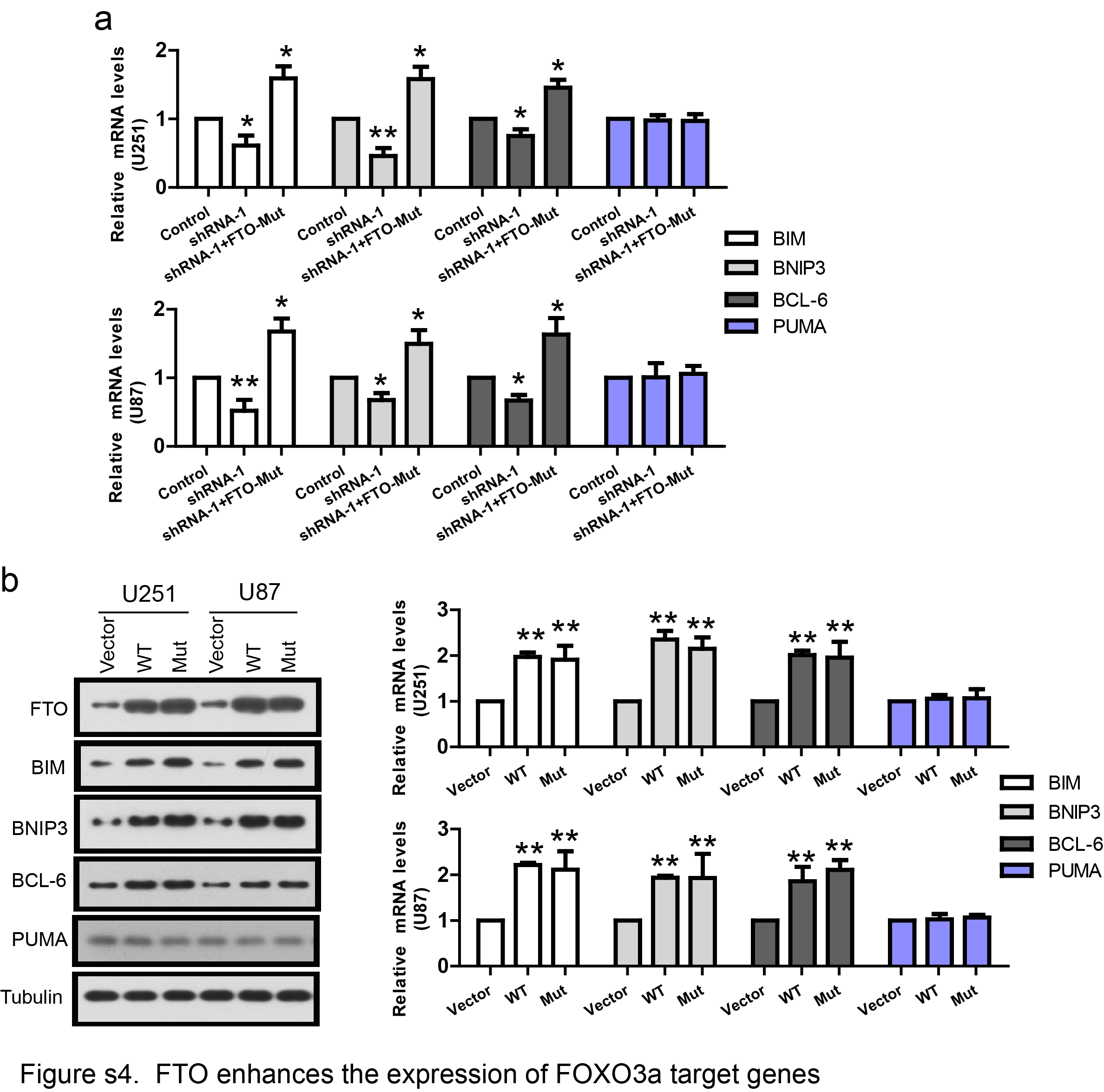


Figure s4. FTO enhances the expression of FOXO3a target genes

(a) qPCR results showing the relative mRNA levels of BIM, BNIP3, BCL-6 and PUMA in U251 and U87 infected with control, FTO shRNA-1 and FTO shRNA-1 plus FTO-Mut. (b) Representative western blots and qPCR results showing the expression of BIM, BNIP3, BCL-6 and PUMA in U251 and U87 cells infected with control, FTO-WT or FTO-Mut. For all, *P<0.05; **P<0.01.


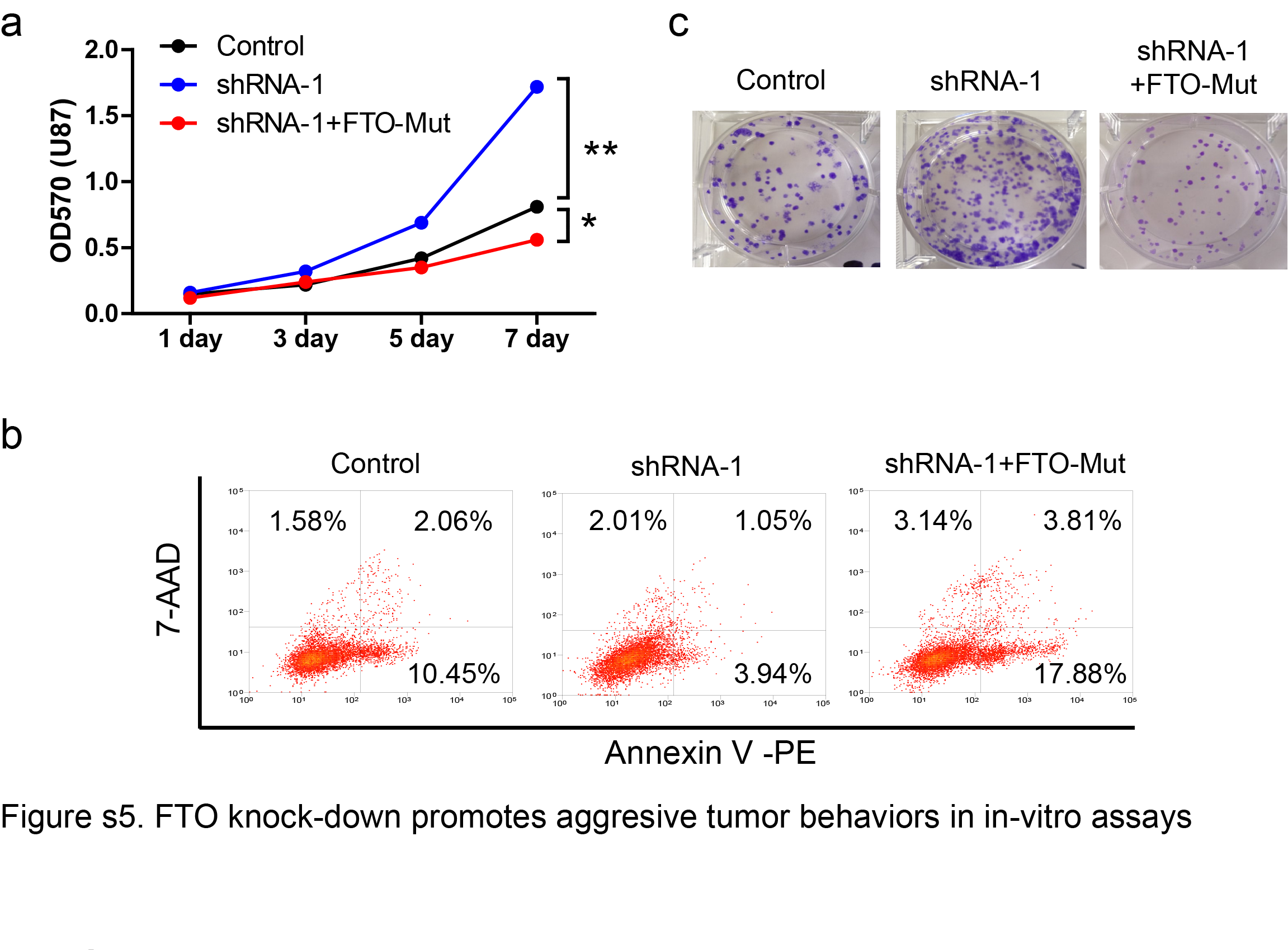


Figure s5. FTO knock-down promotes aggressive tumor behaviors in in-vitro assays

(a) MTT assay showing the growth curves of U87 infected with control, FTO shRNA-1 and FTO shRNA-1 plus FTO-Mut over-expression. (b) Representative results of Annexin V-PE apoptosis assay in U87 infected with control, FTO shRNA-1 and FTO shRNA-1 plus FTO-Mut over-expression. (c) Representative images of colony formation of U87 infected with control, FTO shRNA-1 and FTO shRNA-1 plus FTO-Mut over-expression.


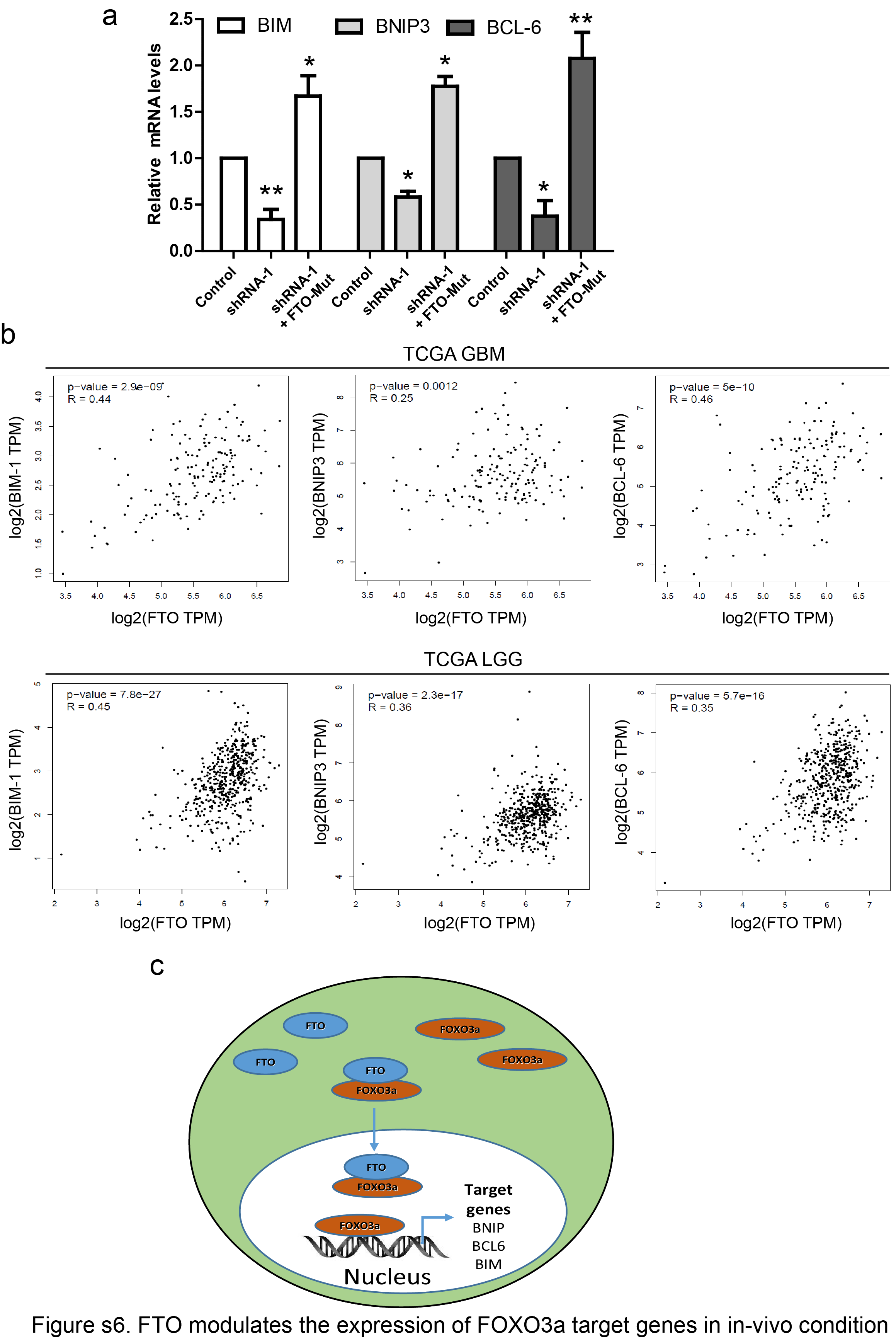


Figure s6. FTO modulates the expression of FOXO3a target genes in in-vivo condition

(a) qPCR results showing the mRNA levels of BIM, BNIP3 and BCL-6 in intracranial glioma tissues infected with control, FTO shRNA-1 or FTO shRNA-1 + FTO-Mut. (b) Scatter plots showing the positive association of FTO expression with BIM, BNIP3 and BCL-6 in TCGA GBM and TCGA LGG datasets. For all, *P<0.05; **P<0.01. (c) Working model showing that FTO plays a tumor-suppressive role in gliomas by interacting with FOXO3a to enhance its nuclear translocation and target gene expression.
